# Mobile health supported multi-domain recovery trajectories after major arthroplasty or spine surgery: a pilot feasibility and usability study

**DOI:** 10.1186/s12891-023-06928-3

**Published:** 2023-10-06

**Authors:** Bhiken I. Naik, Marcel E. Durieux, Rebecca Dillingham, Ava Lena Waldman, Margaret Holstege, Zunaira Arbab, Siny Tsang, Quanjun Cui, Xudong Joshua Li, Anuj Singla, Chun-Po Yen, Lauren K. Dunn

**Affiliations:** 1 Department of Anesthesiology and Neurological Surgery, University of Virginia, Charlottesville, VA USA; 2 Department of Anesthesiology, University of Virginia, Charlottesville, VA USA; 3 Division of Infectious Diseases, Martha Jefferson Hospital, Charlottesville, VA USA; 4 Department of Psychiatry and Neurobehavioral Sciences, University of Virginia, Charlottesville, VA USA; 5 Department of Orthopedics, University of Virginia, Charlottesville, VA USA; 6 Department of Neurological Surgery, University of Virginia, Charlottesville, VA USA

**Keywords:** Recovery trajectories, Functional outcomes, Pain, Psychosocial

## Abstract

**Background:**

Recovery after surgery intersects physical, psychological, and social domains. In this study we aim to assess the feasibility and usability of a mobile health application called PositiveTrends to track recovery in these domains amongst participants undergoing hip, knee arthroplasty or spine surgery. Our secondary aim was to generate procedure-specific, recovery trajectories within the pain and medication, psycho-social and patient-reported outcomes domain.

**Methods:**

Prospective, observational study in participants greater than eighteen years of age. Data was collected prior to and up to one hundred and eighty days after completion of surgery within the three domains using PositiveTrends. Feasibility was assessed using participant response rates from the PositiveTrends app. Usability was assessed quantitatively using the System Usability Scale. Heat maps and effect plots were used to visualize multi-domain recovery trajectories. Generalized linear mixed effects models were used to estimate the change in the outcomes over time.

**Results:**

Forty-two participants were enrolled over a four-month recruitment period. Proportion of app responses was highest for participants who underwent spine surgery (median = 78, range = 36–100), followed by those who underwent knee arthroplasty (median = 72, range = 12–100), and hip arthroplasty (median = 62, range = 12–98). System Usability Scale mean score was 82 ± 16 at 180 days postoperatively. Function improved by 8 and 6.4 points per month after hip and knee arthroplasty, respectively. In spine participants, the Oswestry Disability Index decreased by 1.4 points per month. Mood improved in all three cohorts, however stress levels remained elevated in spine participants. Pain decreased by 0.16 (95% Confidence Interval: 0.13–0.20, *p* < 0.001), 0.25 (95% CI: 0.21–0.28, *p* < 0.001) and 0.14 (95% CI: 0.12–0.15, *p* < 0.001) points per month in hip, knee, and spine cohorts respectively. There was a 10.9-to-40.3-fold increase in the probability of using no medication for each month postoperatively.

**Conclusions:**

In this study, we demonstrate the feasibility and usability of PositiveTrends, which can map and track multi-domain recovery trajectories after major arthroplasty or spine surgery.

## Introduction

Major joint arthroplasty and spine surgery are commonly performed orthopedic procedures worldwide [1, 2]. Recovery after surgery is a complex interplay between physical, psychological, and social factors. With an increasing number of these procedures being performed in older patients with multiple associated physical and psycho-social comorbidities, creating a roadmap of multi-domain postoperative recovery trajectories is important from both a patient and provider perspective [3].

Mobile health (mHealth) applications are increasingly used to manage acute and chronic health diseases. Two mHealth platforms called PositiveLinks and HOPE for management of patients with HIV and opioid use disorder respectively have been developed at our institution. The apps facilitated better medication and appointment adherences by providing reminders and other support to patients. PositiveLinks and HOPE were found to be associated with improved CD4 counts and viral suppression in HIV patients and increased retention of care in opioid treatment programs respectively [4–6].

We used the aforementioned apps as a model framework to develop PositiveTrends, a multi-domain mHealth platform where participants could input functional, psycho-social, pain and prescription medication use data prior to and after surgery. Several studies have demonstrated that postoperative recovery is not uniform, with patients following multiple, different recovery pathways [7, 8]. Predicting the probable recovery trajectory prior to surgery provides an opportunity for patient-centric postoperative management and early identification of deviations from these recovery trajectories.

In this pilot study our primary aim was to assess feasibility and usability of PositiveTrends to collect patient-reported outcomes, psycho-social, pain and prescription medication data after arthroplasty and spine surgery. Our secondary aim was to generate procedure-specific, multi-domain recovery trajectories. We hypothesized that PositiveTrends would be feasible, usable, and acceptable to participants.

## Methods

### Study inclusion and exclusion criteria

We recruited participants greater than 18 years of age undergoing hip or knee arthroplasty or spine surgery, who spoke English and owned a personal smartphone. Exclusion criteria included pregnancy, prisoners, or participants unable to provide consent. Participants were recruited from November 2021 to March 2022. Participants were followed for a maximum of 180 days after the index procedure.

### Study protocol

Convenience sampling was used to recruit participants prior to surgery. After obtaining informed consent the research assistant downloaded PositiveTrends and installed it on the participants’ personal smartphone. In-person orientation on app usage was provided, and a quick start guide was given to all participants. Summary of the study protocol and timeline for study participants is reported in Table 1.

**Table 1:** Study Protocol and Timeline. KOOS-12-Knee injury and Osteoarthritis Outcome Score-12, HOOS-12-Hip disability and Osteoarthritis Outcome Score-12, MOS-Medical Outcomes Study

	Visit 1 Preoperative	Daily	Visit 2	Visit 3	Visit 4
**Study Day**	0	1-180	30	90	180
Informed Consent	X				
Review of medical information	X				
Install PositiveTrends on smartphone	X				
Orientation to PositiveTrends	X				
Oswestry Disability Index	X			X	X
HOOS-12 OR KOOS-12 Knee Survey	X			X	X
MOS Social Support Survey	X			X	X
Daily Check-In: Mood, Stress, Pain and Prescription Medication Use		X			
System Usability Scale (SUS)			X		X
Delete app from smartphone					X

### Study variables

Demographic, surgery type, primary vs. revision procedure data was collected [9].

### Positivetrends Application features

#### App security and data privacy

PositiveTrends is a mobile health service that has a linked web-based portal and mobile health-based application. The web portal is accessed using a secure password by study coordinators to enter demographic and surgical data of study participants who consented to be in the study and provide participant access to PositiveTrends. The mobile application was available on Android and iOS.

Encryption requirements.

All mobile data was transmitted over HTTPS/SSL (TLSv1.1,TLSv1.2) SSL ciphers: EECDH + AESGCM:EDH + AESGCM:AES256 + EECDH:AES256 + EDH with session timeout of 10 min. Data was stored encrypted with Amazon Web Services Key Management Service (AWS KMS).

Access to app data.

Study coordinators were able to view study participants’ data. Study participants were able to view and modify their data entry in the app. All data access was logged and stored in multiple locations including the local study computer and the cloud.

App and system updates.

PositiveTrends technical support performed routine maintenance on the app service including updated to the web portal or mobile applications.

## Outcomes

### Primary outcome

#### Positivetrends feasibility assessment

The feasibility of PositiveTrends as a tool to assist recovery after arthroplasty and spine surgery was evaluated by how engaged participants were with the app through the study period. This was computed as the number of inputs or responses by participants in response to the app notifications to complete pre-defined task (number of participant responses/ number of app notifications). Proportion of response rates for the daily, 1, 3 and 6-month app notifications were recorded and summarized.

#### Positivetrends usability assessment

System Usability Scale (SUS) was used to assess the usability of PositiveTrends. SUS is a 10-item survey to assess app usability in which the participants rate their responses on a 5-point Likert-type scale (*1 = strongly disagree, 5 = strongly agree*) [10]. To calculate the SUS score, we first added the score contributions from each item, after recoding reverse scored items and then multiplied the sum of the scores by 2.5 to obtain the overall value of SUS. SUS scores ranged between 0 and 100, with higher scores representing better usability. The threshold usability score is ≥ 68. SUS was assessed at two time points: days 30 and 180 after surgery [10]. Cronbach’s alpha of the SUS at day 30 was 0.86, reflecting high internal consistency. Participants also had an opportunity to add text comments regarding app usability after completing the SUS survey.

### Secondary outcomes

#### Participant reported outcomes

Functional status measures included the Knee injury and Osteoarthritis Outcome Score-12 (KOOS-12), Hip disability and Osteoarthritis Outcome Score-12 (HOOS-12) and the Oswestry Disability Index (ODI) for knee, hip, and spine surgery participants respectively.

The HOOS-12 contains 12 questions, scored from 0 to 4 points, with 0 representing no hip problems and 4 representing extreme hip problems, for the individual questions. HOOS-12 scale scores was transformed so 0 is the worst possible and 100 is the best possible score [11]. KOOS-12 contains four KOOS Pain items, four KOOS Function (Activities of Daily Living and Sport/Recreation) items, and four KOOS Quality of Life items. Each item is scored from 0 to 4, with 0 representing no knee problems and 4 representing extreme knee problems. KOOS-12 scale scores was transformed so 0 is the worst possible and 100 is the best possible score [11, 12]. Due to a technical issue, we could only extract the pain and daily living function subscales for the KOOS-12 and HOOS-12. Therefore, only pain and function subscales for the KOOS-12 and HOOS-12 are reported in the current study, and no summary impact scores are computed [12]. All subscales showed acceptable to high internal consistency (Cronbach’s alpha = 0.71, 0.83, 0.86, and 0.94 for HOOS-12 pain, HOOS-12 function, KOOS-12 pain, and KOOS-12 function subscale, respectively) at baseline.

The ODI consists of ten items. For each item, the patient selected the statement (out of six) that most closely describes their current condition. Each item has a maximum possible score of 5, with 0 being the first statement selection, and 5 being the sixth statement selection. A total ODI score was computed by summing the scores on each item (maximum possible total score = 50). The total ODI score was then categorized as follows: 0–4: no disability, 5–14: mild disability, 15–24: moderate disability, 25–34: severe disability, 35–50: completely disabled. The ODI showed high internal consistency (Cronbach’s alpha = 0.83) at baseline.

Participant-reported baseline measures were entered into PositiveTrends under supervision of the research coordinator in the clinic prior to surgery. At 90 and 180 days after surgery participants received a notification with repeated reminders in PositiveTrends to complete the follow-up functional measures.

### Psycho-social measures

#### Mood and stress

The mood and stress measures utilized in this study were previously used and validated in the Positivelinks and HOPE mobile health application, from which PositiveTrends was based [6, 13]. Daily app notifications for participants to report their mood and stress started the day of surgery and continued to 180 days after the index procedure. Participants could respond immediately to the notification or retrospectively report their mood and stress for the prior two days, if they missed or failed to respond to earlier app notifications. Mood was reported on one item with five response categories (*Very Happy, Happy, OK, Sad, Very Sad*); each category is associated with a weather-themed emoji. Stress was reported on one item with five response categories (*Very Low, Low, Medium, High, Very High*); each category is associated with a colored box emoji from green to red. To facilitate data analysis, the visual scales for mood and stress were converted to a 5-point Likert-type scale (1 = worst mood/lowest stress, 5 = best mood/highest stress). The mood and stress scale were analyzed as continuous variables.

### Social support

The Medical Outcomes Study (MOS) Social Support Survey was administered via the app before surgery in the preoperative clinic, 90 and 180-days after surgery. The MOS is a 19-items survey composed of 4 subscales (8 items in emotional/informational support, 4 items in tangible support, 3 items in affectionate support, 3 items in positive social interaction) [14]. An average score was computed for each subscale, and an overall index was computed by taking the mean of all 19 items. Subscale scores were transformed as follows: 100 x (mean subscale score − 1)/(5 − 1). The overall index score was transformed as follows: 100 x (observed score − minimum possible score)/(maximum possible score − minimum possible score) such that the scores range from 0 to 100, with higher scores reflecting more support. The MOS showed high internal consistency (Cronbach’s alpha = 0.97, 0.96, 0.87, 0.96, and 0.93 for the overall scale, emotional/informational support, tangible support, affectionate support, and positive social interaction subscales, respectively) 90 days after surgery.

### Pain and medication usage

Daily pain and prescribed medication use was collected with daily app notifications. Pain was reported on a 4-point visual ordinal analogue scale (*0 = No Pain, 1 = Mild Pain, 2 = Moderate Pain, 3 = Severe Pain*) with an appropriate emotional face emoji associated with each response category. There is high concordance (0.76–0.92) between the traditional 11-point numerical rating scale and a visual analogue scale [15–17].

Prescribed medication use was assessed using four response categories (*As Prescribed, More than prescribed, Less than prescribed, None*), with each response category having an appropriate, associated colored pie chart.

### Statistical analysis

Continuous variables were summarized with means, standard deviations, medians, and interquartile ranges, where appropriate. Categorical variables were summarized with counts and percentages.

We used generalized linear mixed effects regression (GLMER) models to estimate the change in the outcome over time. Postoperative time (months) was included as fixed effects, and participants were included as random effects to consider within-participant correlations. Linear mixed effects regression models were performed for continuous or Likert-type outcome variables, whereas GLMERs with a logistic link were performed for binary outcome variables. For outcome variables specific to the surgery (HOOS-12, KOOS-12 and ODI) and participant self-reported outcomes (stress, mood, pain, and medication use), GLMER models were performed separately for participants who underwent hip, knee, and spine surgery. For general outcomes (SUS and MOS), initial analyses showed no substantial differences across the three cohorts; therefore, participants were collapsed across the surgery types for more parsimonious models. All statistical analysis was performed in R 4.1.2 (the R Foundation) [18].

## Results

A total of 132 spine, hip, and knee surgery patients were screened for participation in the study over a total of 18 weeks. Patients were not eligible for the following reasons: research coordinator scheduling conflicts (n = 40), lack of smartphones (n = 11), not interested in participating in research or sharing personal information (n = 27), overlapping studies (n = 4), participant not comfortable with smartphone applications (n = 5). A total of 45 participants were recruited and consented over a four-month recruitment period. After recruitment, participants were excluded due to an incorrect procedure (n = 1) and the surgery being canceled (n = 2). The final cohort enrolled 42 participants. Demographic and clinical data for the three surgical cohorts are reported in Table 2. Half of the participants in this cohort underwent spine surgery. Most of the arthroplasty procedures were primary procedures, while 24% of the spine procedures were revision procedures.

**Table 2:** Demographic and Clinical Characteristics. Data reported as mean and standard deviation, count and percent or median and interquartile range. SD-standard deviation, n-number, IQR-interquartile range

Variable	Hip Arthroplasty (N = 8)	Knee Arthroplasty (N = 13)	Spine Surgery (N = 21)
Age, years, mean ± SD	61 ± 12	66 ± 11	57 ± 15
Sex, male, n (%)	3 (38)	3 (23)	9 (43)
Body Mass Index (kg/m^2^), mean ± SD	28 ± 7.6	31 ± 5.7	30 ± 8.3
**Race**			
White, n (%)	7 (87.5)	12 (85.7)	17 (80.9)
Black, n (%)	1 (12.5)	1 (14.3)	3 (14.3)
Alaskan/Native American, n (%)	0	0	1 (4.8)
Primary Procedure, n (%)	8 (100)	12 (85.7)	16 (76)

### Primary outcome

#### Feasibility

**Fig. 1:**
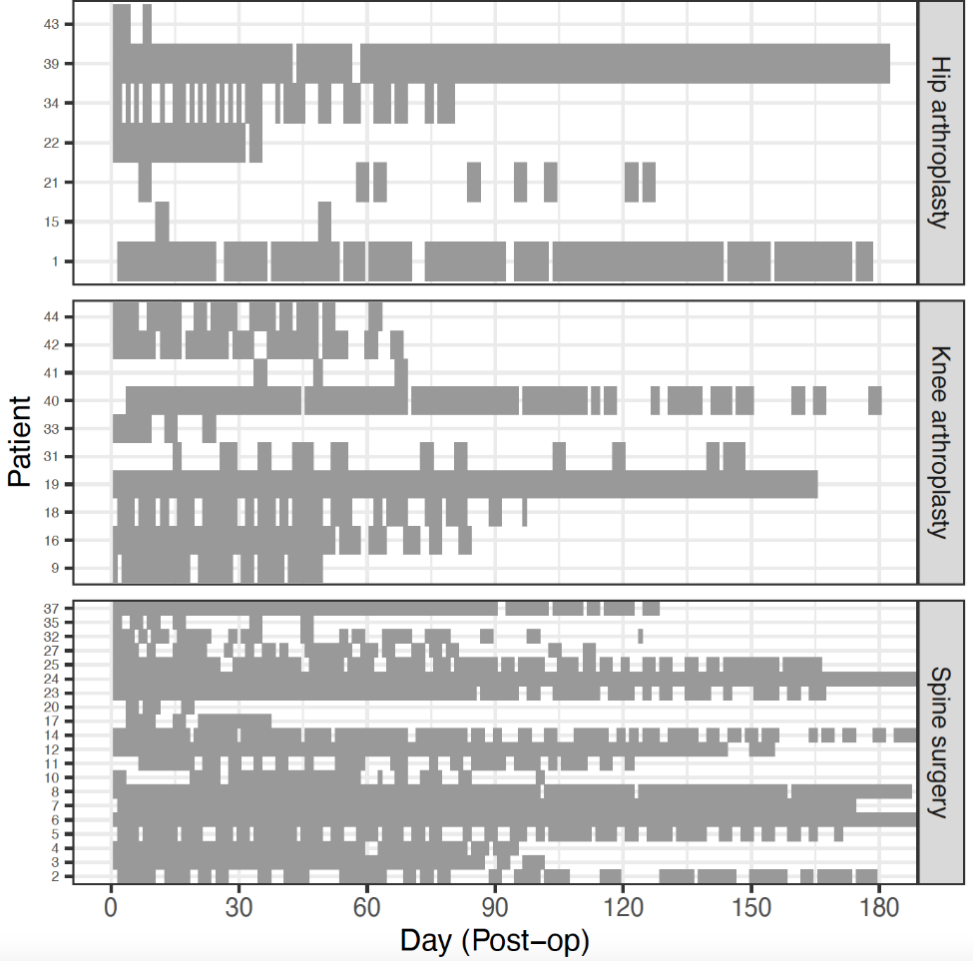
PositiveTrends usage over time in the three surgical cohorts. Grey tiles represent app completion missing tiles indicate no response by participants

PositiveTrends usage over time is plotted in Fig. 1. Out of the 180 follow-up days postoperatively, the proportion of app responses was highest for participants who underwent spine surgery (median = 78, range = 36–100), followed by those who underwent knee arthroplasty (median = 72, range = 12–100), and hip arthroplasty (median = 62, range = 12–98). Amongst participants undergoing arthroplasty procedures, app engagement decreased over time.

#### System usability scale

The median (interquartile range) for SUS score was 86 (54–95) at 30 days post-operatively, and 82 82 (75–95) at 180 days postoperatively. Within the cohort 64% (n = 23) and 83% (n = 24) had SUS score > = 68 at month 1 and 6, respectively. There was no statistically significant change in SUS scores between the two time points (*b* = 6.93, 95% CI = -3.7–17.6, *p* = 0.197).

### Secondary outcomes

#### Participant reported outcomes

**Fig. 2:**
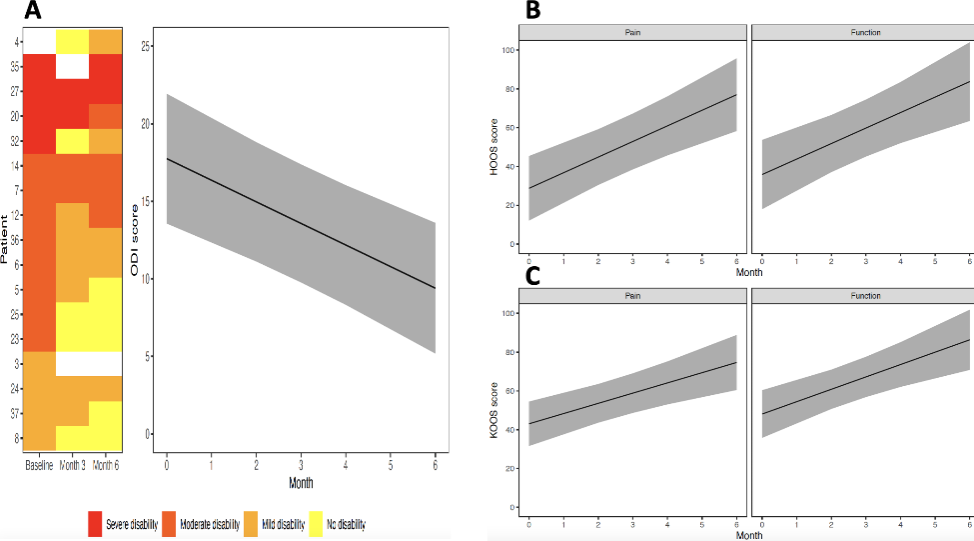
Participant level heat map and estimated change in Oswestry Disability Index scores over time (panel A), estimated changes in Pain and Function HOOS score (panel B) over time and estimated changes in Pain and Function KOOS score over time (panel C)

Most participants who underwent spine surgery had moderate (50%; red tiles) or severe disability (25%; orange tiles) at baseline, whereas most participants had mild (31%; tan tiles) or no disability (31%; yellow tiles) by six months (Fig. 2, panel A). Of the four participants with severe disability (red tiles) at baseline, half of them showed improvement to moderate (orange tiles) or mild disability (tan tiles). These observations are consistent with results from the statistical models, ODI decreased by 1.4 points (95% CI: 2.0–0.80, *p* < 0.001) points per month after spine surgery (Fig. 2, panel A).

There was a significant improvement in pain and function subscales over time for both HOOS-12 and KOOS-12 (Fig. 2, panel B and C). For participants who underwent hip arthroplasty, pain and function improved by 8 points (95% CI: 4.5–11.5, *p* < 0.001) and 8 points (95% CI: 3.9–12.1, *p* = 0.001) respectively each month after the surgery (Fig. 2, panel B). For participants who underwent knee arthroplasty, pain and function improved by 5.3 points (95% CI: 2.6–7.9, *p* < 0.001) and 6.4 points (95% CI: 3.2–9.5, *p* < 0.001) points per month after surgery (Fig. 2, panel C).

#### Psycho-social features

**Fig. 3:**
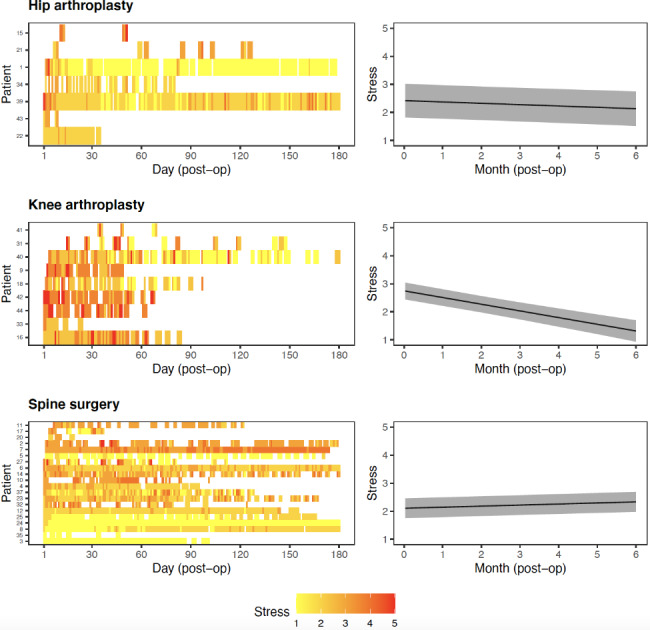
Participant level heat map (left panel) and estimated change in stress over time (right panel)

Participants’ self-reported stress levels are illustrated in (Fig. 3, left panel). Most participants who underwent hip or knee arthroplasty reported decreasing levels of stress (from red to orange/yellow tiles) across time, however the decrease in stress levels was more pronounced for participants who underwent knee arthroplasty (0.21-point decrease per month, 95% CI = 0.01–0.08, *p* = 0.009) than those who underwent hip arthroplasty (0.05-point decrease per month, 95% CI = 0.18–0.29, *p* < 0.001). However, the changes in stress levels were mixed for participants who underwent spine surgery, with most participants reporting an increase in stress (0.04-point increase per month, 95% CI = 0.02–0.05, *p* < 0.001), even 90 days postoperatively. These changes are illustrated in Fig. 3 (right panel).

**Fig. 4:**
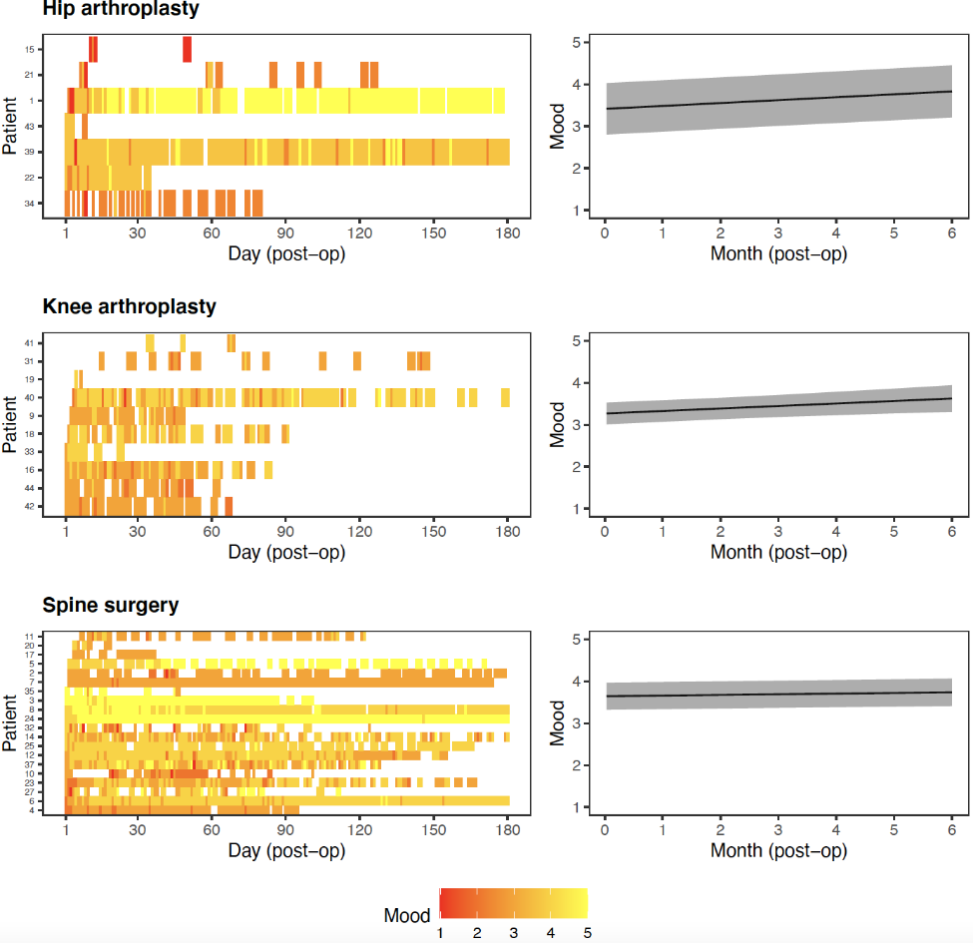
Participant level heat map (left panel) and estimated change in mood over time (right panel)

Figure 4 (left panel) depicts participants’ self-reported mood levels. In the first month postoperatively, most participants reported being *sad or OK* (2–3 points). By three months postoperatively, participants were more likely to report being *OK or happy* (3–4 points). Consistent across the hip (0.07-point increase per month, 95% CI: 0.04–0.09, *p* < 0.001), knee (0.06-point increase per month, 95% CI: 0.02–0.1, *p* = 0.005) and spine (0.02-point increase per month, 95% CI: 0–0.03, *p* = 0.021) cohorts, there was a significant increase in mood levels over time. However, the effect was relatively small (< 1 point increase per month), as reflected in Fig. 4 (right panel).

### Social support

Emotional/informational (0.17 point change per month, 95% CI: -1.3–1.6, *p* = 0.814), Tangible (0.1 point change per month, 95% CI: -1.2–1.4, *p* = 0.847), Affectionate (-0.73 point change per month, 95% CI: -1.8–0.3, *p* = 0.158), Positive social interaction (0.02 point change per month, 95% CI: -1.2–1.2, *p* = 0.976) and Overall score (0.04 point change per month, 95% CI: -1.0–1.1, *p* = 0.939) demonstrated no substantial change over time.

#### Pain and medication

**Fig. 5:**
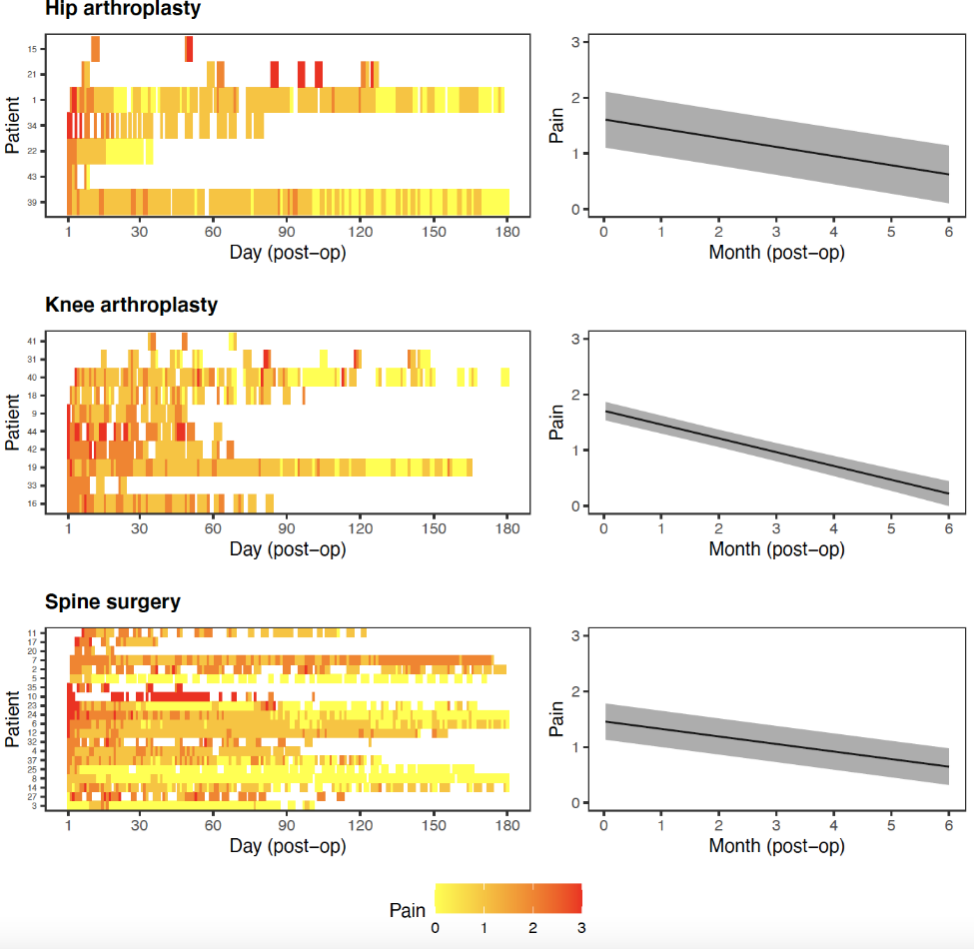
Participant level heat map (left panel) and estimated change in pain over time (right panel)

Pain reported by participants over the six months period is illustrated in Fig. 5 (left panel). Self-reported pain levels vary across participants, though most participants reported lower levels of pain (0 or 1 unit) over time. Results showed a statistically significant decrease in pain levels for hip (0.16-point decrease per month, 95% CI: 0.13–0.20, *p* < 0.001), knee (0.25-point decrease per month, 95% CI: 0.21–0.28, *p* < 0.001) and spine (0.14-point decrease per month, 95% CI: 0.12–0.15, *p* < 0.001) participants over time. There was a 0.14-to-0.25-point reduction in pain levels per month after the surgery across the three surgical cohorts (Fig. 5, right panel).

**Fig. 6:**
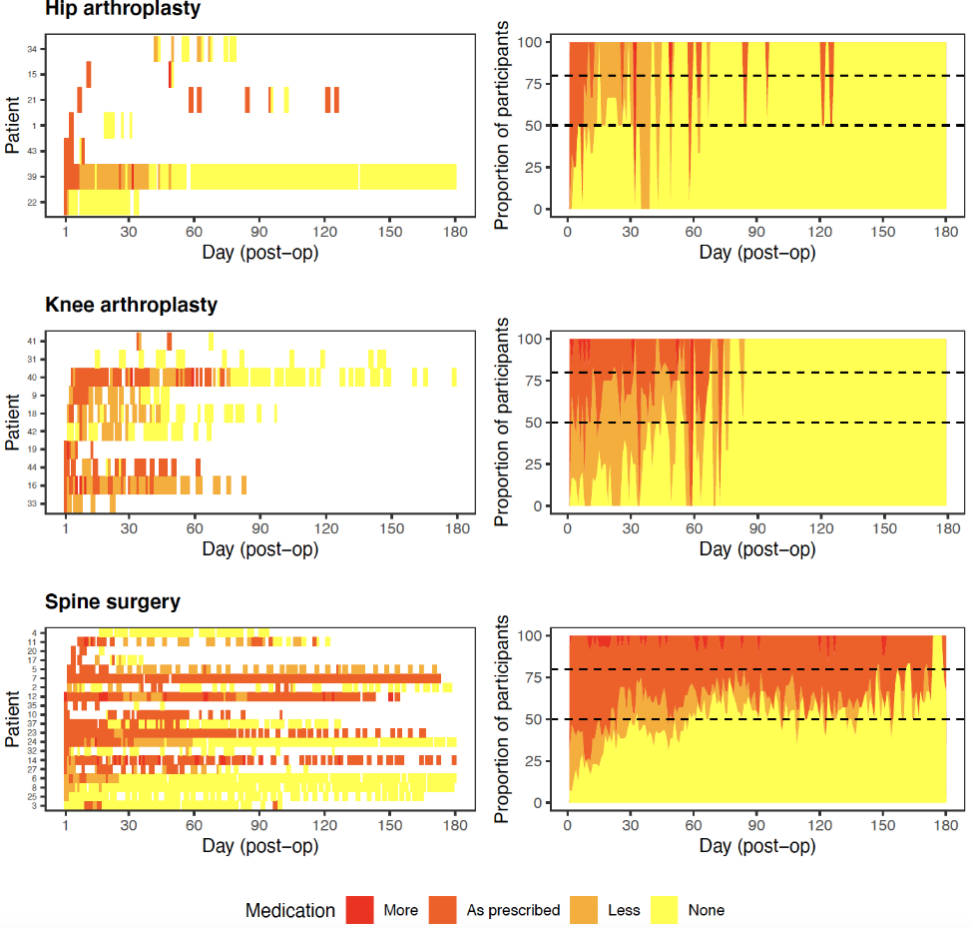
Participant level heat map (left panel) and proportion of participants with different prescribed medication use (right panel)

As demonstrated in Fig. 6 (left panel), self-reported use of prescribed pain medication after surgery varied by participant across time. Some participants reported less use of pain medications over time, whereas others reported relatively stable use of pain medication. At least 50% of hip or knee arthroplasty participants reported not using medication by 3.5 months after surgery (Fig. 6, right panel). In contrast, most spine surgery participants continued to report using some pain medication until at least 5 months postoperatively.

**Fig. 7:**
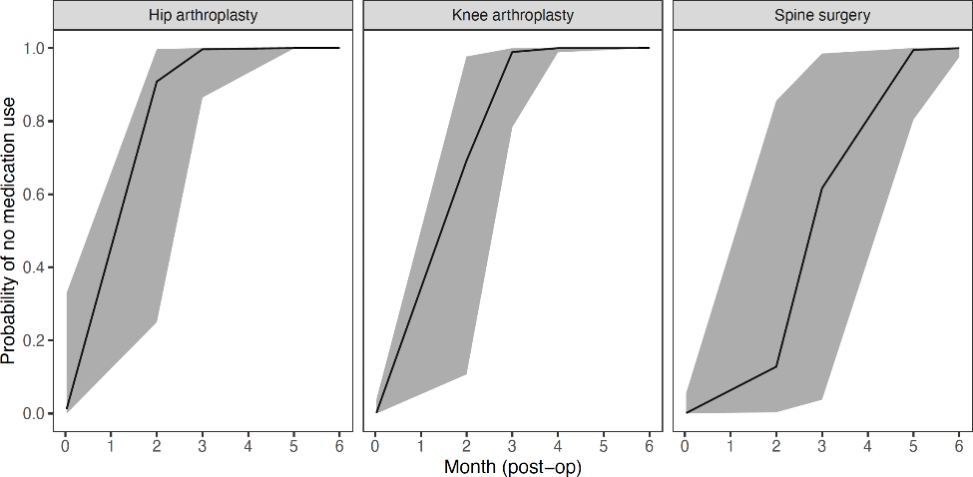
Estimated probability of using no medication over time. The shaded area represents the 95% confidence intervals of the estimated effects

In the hip (Odds Ratio [OR]: 29.5, 95% CI: 9.1–95.7, *p* < 0.001), knee (OR: 40.3, 95% CI: 15.4–105.2, *p* < 0.001) and spine (OR: 10.9, 95% CI: 7.4–16.1, *p* < 0.001) participants, the odds of using no medication increases for each month postoperatively. There was a 10.9-to-40.3-fold increase in the probability of using no prescribed pain medication for each month postoperatively. Participants who underwent hip or knee arthroplasty were likely to reduce pain medication use to none by three months after surgery (Fig. 7). However, the confidence intervals were very large due to the large amount of missing data over time, suggesting a large variability across participants.

## Discussion

Our study demonstrates the feasibility and usability of PositiveTrends, amongst study participants to record and track procedure-specific, multi-domain recovery trajectories after major arthroplasty or spine surgery. We demonstrated higher, sustained app engagement in the spine surgery cohort, compared to the knee and hip arthroplasty group over the study period. This finding was not surprising considering the longer recovery times and higher incidence of chronic pain in patients undergoing spine surgery. Our app demonstrated high SUS scores at 30 and 180 days postoperatively.

Patient reported functional recovery trajectories and mood demonstrated sustained improvement amongst all three surgical cohorts, however stress increased over time amongst participants undergoing spine surgery. Future studies are required to understand factors associated with the worsening stress phenotype seen in the spine cohort.

An additional important finding of our study was the different patterns of pain medication discontinuation seen between the arthroplasty and spine cohorts. Patients undergoing major arthroplasty or spine surgery are at risk of developing prolonged pain and opioid use disorder after surgery [19, 20]. Furthermore, psycho-social features such as anxiety, depression and catastrophizing have been associated with increased postoperative opioid use and lower quality of recovery [21]. Findings from our study are clinically relevant and can provide care teams and patients with contemporaneous visual data about characteristic pattern, direction, trajectory and potential deviations from expected recovery trajectories. This can facilitate early goal-directed interventions such as referral to addiction medicine specialist, to reduce the risk of prolonged opioid use. Recovery trajectories in the pain and medication use domain can be used as a visual aid with patients as part of a shared decision model prior to surgery, regarding the amount of opioid to be prescribed after surgery. Studies have demonstrated that using this approach results in significantly less opioid use after surgery [22–24].

Future developments to the PositiveTrends app will include overlaying participant reported data on the procedure-specific recovery trajectory, providing participants a visual guide of their postoperative progress. In addition, as more participants are recruited, tuning of the model based on preoperative risk predictors will be performed, allowing for a more ‘personalized’ recovery trajectory to be projected. Finally, based on input from users, future modifications of opioid use choices, decreasing the frequency of check-ins and more free text options might be considered.

Our study has several limitations. Unfortunately, due to a technical issue, quality of life data could not be obtained for the KOOS-12 and HOOS-12. Recovery trajectory will need to be updated in future studies with PositiveTrends for this functional subcomponent. Secondly, this was a feasibility study. Due to our small sample size, we do not have sufficient power to examine whether domain-specific recovery trajectories differ for subgroups of patients. For example, we had a heterogeneous group of spine surgery procedures, ranging from minimally invasive decompressions to multi-level fusions. As patient recruitment increases, we will be able to apply more advanced statistical models to investigate and compare potential differences in recovery trajectories across heterogeneous groups of patients, as well as examine risk factors that may potentially affect recovery trajectories.

In conclusion, in this pilot study we demonstrate the feasibility and usability of a mHealth platform to map and track multi-domain postoperative recovery trajectories after major arthroplasty or spine surgery. The findings of this study support implementation of PositiveTrends in a larger, heterogenous cohort of participants.

## Data Availability

The data presented in this study are available on request from the corresponding author. The data are not publicly available.

## Abbreviations

12 (KOOS-12): Knee injury and Osteoarthritis Outcome Score
12 (HOOS-12): Hip disability and Osteoarthritis Outcome Score
ODI: Oswestry Disability Index
MOS: Medical Outcomes Study
SUS: System Usability Scale
GLMER: Generalized linear mixed effects regression
